# An Empty Scoping Review on the Roles of mHealth Interventions on Menstrual Hygiene in Africa: Implications for Sexual and Reproductive Health Research, Policy, and Practice

**DOI:** 10.1002/hsr2.72026

**Published:** 2026-03-04

**Authors:** Kafayat Aminu, Precious Chika Nnannah, Oluwatobi Emmanuel Adegbile, Adetayo Olorunlana, Yovanthi Anurangi Jayasinghe, Ugochukwu Anthony Eze, Afeez Abolarinwa Salami, Emeka Benjamin Okeke, Olubukola Omobowale, Michael Renfrew, Rita Amarachi Nwebo, Kehinde Kazeem Kanmodi

**Affiliations:** ^1^ Centre for Evidence Synthesis and Implementation Research Cephas Health Research Initiative Inc Ibadan Nigeria; ^2^ Centre for Digital Health Research, Innovation and Practice Cephas Health Research Initiative Inc Ibadan Nigeria; ^3^ Centre for Disease Control and Prevention Cephas Health Research Initiative Inc Ibadan Nigeria; ^4^ Scientific Advisory Board Cephas Health Research Initiative Inc Ibadan Nigeria; ^5^ College of Health Sciences Caleb University Imota Nigeria; ^6^ Department of Research and Innovation University of Technology and Entrepreneurship Phnom Penh Cambodia; ^7^ Department of Community Medicine University of Ibadan Ibadan Nigeria; ^8^ Department of Research University of Puthisastra Phnom Penh Cambodia; ^9^ School of Health and Life Sciences Teesside University Middlesbrough UK; ^10^ Department of Public Health Thomas Adewumi University Oko Nigeria

**Keywords:** Africa, menstrual hygiene, mHealth, review

## Abstract

**Background and Aims:**

Menstrual hygiene remains a critical public health issue in Africa, where many women and girls face inadequate water, sanitation, and hygiene (WASH) facilities, limited access to menstrual products, and persistent social stigma. Mobile health (mHealth) interventions have been effective at addressing systemic issues in other health domains, yet their role in improving menstrual hygiene in African contexts remains unclear. This scoping review aimed to identify and map empirical evidence on mHealth interventions for menstrual hygiene in Africa, assess reported impacts, and highlight research and policy gaps.

**Methods:**

This scoping review followed the Arksey and O'Malley framework and adhered to the PRISMA‐ScR reporting guidelines. A comprehensive search of nine databases was conducted to retrieve all relevant studies published from inception to April 7, 2025. Eligible studies were to be empirical, peer‐reviewed studies conducted in Africa, and published in English. Screening and selection were undertaken independently by two reviewers, with conflicts resolved by consensus. Data extraction, collation, and summarization could not be done as no study was found eligible for inclusion into this review.

**Results:**

A total of 687 records were retrieved; however, none of them met the inclusion criteria, primarily due to non‐relevance of the population, context, or outcomes of interest. Hence, this scoping review is empty.

**Conclusions:**

The absence of published empirical research on mHealth interventions for menstrual hygiene in Africa highlights a critical knowledge gap. Considering the significant menstrual health challenges across the continent and the proven potential of mHealth in other public health areas, this represents a missed opportunity for policy intervention and public health innovation. There is an urgent need for context‐specific research, community engagement, and multisectoral collaboration to design, implement, and evaluate mHealth strategies that address menstrual hygiene needs in Africa, informing both local and global sexual and reproductive health policies.

## 1 Introduction

Globally, menstrual hygiene affects approximately 1.8 billion women and girls of reproductive age, with significant challenges persisting in low and middle‐income countries [1]. Menstrual hygiene is a critical public health issue, particularly in Africa, where an estimated 70% women and girls face challenges in accessing water, sanitation, and hygiene (WASH) facilities, and suitable menstrual products; further complicated by a lack of comprehensive menstrual education [2, 3, 4]. Poor management of menstrual hygiene is associated with adverse outcomes such as school absenteeism, social stigma, and increased risk of infections [3]. Addressing these challenges is essential to improving the health, dignity, and empowerment of women and girls across the continent.

Mobile health (mHealth) intervention is defined as the use of mobile devices to deliver health information and services and is increasingly being explored as innovative solution to bridge gaps in healthcare access [5, 6]. mHealth tools have demonstrated potential in addressing several public health challenges associated with healthcare transitions by providing education, improving accessibility to services, and influencing behavioral change [7, 8]. In the context of menstrual hygiene, mHealth interventions have the potential to play a transformative role by offering culturally sensitive education, facilitating access to menstrual products, and reducing stigma through community engagement [9, 10].

Despite the promise of mHealth tools, the integration of these technologies into menstrual hygiene initiatives remains underexplored in Africa. Barriers such as limited digital literacy, inadequate infrastructure, and sociocultural norms could hinder their adoption and effectiveness [3, 9]. While several mHealth solutions have been piloted globally, such as period‐tracking apps and SMS‐based educational platforms, their scalability and impact on menstrual hygiene outcomes in African contexts require further investigation [11, 12].

Empirically, menstrual health has been a neglected area of research and policy focus in many African countries. As cultural taboos surrounding menstruation have perpetuated misinformation and stigma, limiting open discussions about menstrual health needs [13]. This has contributed to a lack of sustainable interventions tailored to the unique challenges faced by women and girls in diverse African settings. Recent efforts to improve menstrual hygiene have included distributing reusable pads, enhancing WASH infrastructure, and providing menstrual education through workshops [14, 15]. However, these interventions often fail to address systemic barriers such as stigma or reach underserved populations effectively [16, 17, 18]. It is posited that, by leveraging mHealth technologies, there is an opportunity to develop scalable and context‐specific solutions that can complement existing efforts while addressing persistent gaps.

The aim of this scoping review is to map existing empirical evidence on the roles of mHealth interventions in improving menstrual hygiene across Africa. We seek to identify core components of effective mHealth strategies, assess their impact on menstrual hygiene outcomes, and provide recommendations for integrating these technologies into broader public health frameworks. Hence, this review seeks to answer the following questions:

### 1.1 Research Question

1. What are the available mHealth solutions (e.g., period‐tracking apps, SMS‐based educational platforms, etc.) for menstrual hygiene in Africa?
2. How effective are the available mHealth solutions for addressing menstrual hygiene in Africa?
3. What are the challenges with acquiring, utilizing, and scaling available mHealth solutions to improve menstrual hygiene in Africa?
4. What are the recommendations for future research, innovations, and effectiveness of mHealth solutions in menstrual hygiene?

## 2 Methods

### 2.1 Title and Protocol Registration

The title and protocol of this scoping review have been registered in the Open Science Framework (https://osf.io/prhv6).

### 2.2 Study Design

This review was guided by the stepwise guidelines recommended by Arksey and O'Malley for conducting scoping reviews [19]. Additionally, we used the Preferred Reporting Items for Systematic Reviews and Meta‐analyses extension for Scoping Reviews guidelines (PRISMA‐ScR) in reporting this review [20].

### 2.3 Review Selection Criteria

In determining studies for inclusion, we used the following inclusion criteria:

1. Empirical studies, of any research design, are published in peer‐reviewed journals as original research articles.
2. Empirical studies investigating the use of mHealth interventions (including mobile health applications, mobile phone‐based short messaging services, and wearable portable devices) on menstrual hygiene among populations in Africa.
3. Empirical studies published in English.
4. Empirical studies whose full texts are accessible.
5. Empirical studies published from inception to date.

However, we excluded case series, case reports, scoping reviews, systematic reviews, letters to the editor, and meta‐analyses.

### 2.4 Search Strategy

To ensure that our search terms or phrases were sensitive and highly selective, the study authors conducted a detailed review of key terms that aptly capture the subject matter under consideration. Next, the research team critically reviewed each recommendation and jointly adopted a unified search strategy, ensuring that each subject search term was robust and closely aligned with standard and medical conventions that corroborate the focus of this review. Subsequently, we conducted a systematic search on April 7, 2025, to extract relevant studies from the listed bibliometric databases using the unified search terms: PubMed, SCOPUS, AMED—The Allied and Complementary Medicine Database, CINAHL Ultimate, Dentistry and Oral Sciences Source, SPORTDiscus with Full Text, APA PsycArticles, Psychology and Behavioral Sciences Collection, and APA PsycInfo.

For the mobile health application subject search, we utilized the following search terms: “Digital” or “GSM” or “text messages” or “SMS” or “MMS” or “mobile phone” or “mobile health” or “mHealth” or “cell phone” or “telecommunications” or “Apps” or “application” or “programme” or “android” or “iPhone” or “smart phone” or “technology” or “tablet” or “tabloid” or “iPad” or “device” or “technology enhanced.”

For the menstrual hygiene subject search, we used the following search terms: “menses” or “menstrua*.”

For the African countries, dependencies, and territories subject search, we utilized the following search terms: “Algeria,” “Angola,” “Benin,” “Botswana,” “Burkina Faso,” “Burundi,” “Cameroon,” “Central African Republic,” “Chad,” “Ivory coast,” “Cote d'Ivoire,” “Djibouti,” “Democratic republic of Congo,” “Egypt,” “Equatorial Guinea,” “Eritrea,” “Eswatini,” “Ethiopia,” “Gabon,” “Gambia,” “Ghana,” “Guinea,” “Guinea‐Bissau,” “Kenya,” “Lesotho,” “Liberia,” “Libya,” “Madagascar,” “Malawi “Mauritania,” “Mauritius,” “Mali,” “Mauritania,” “Morocco,” “Mozambique,” “Namibia,” “Niger,” “Nigeria,” “Rwanda,” “Sao Tome and Principe,” “Senegal,” “Seychelles,” “Sierra Leone,” “Somalia,” “South Africa,” “Sudan,” “Tanzania,” “Togo,” “Tunisia,” “Uganda,” “Zambia,” “Zimbabwe,” “Reunion,” “Saint Helena,” “Western Sahara,” “Mayotte.” The search outcomes are presented in Supporting Information S1: Tables S1–S3.

### 2.5 Study Selection

**Figure 1:**
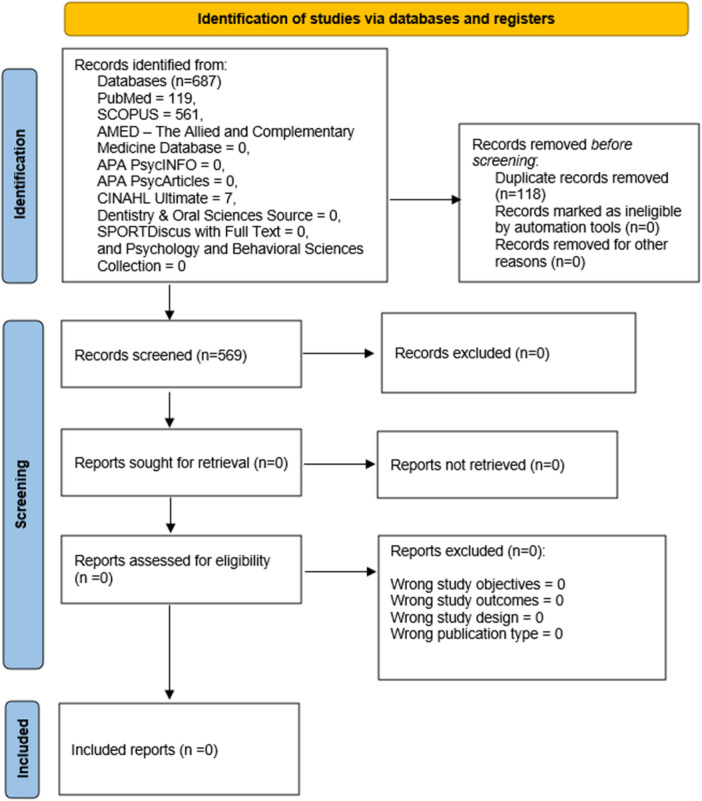
PRISMA 2020 flowchart detailing the systematic search and screening process of the scoping review, which resulted in the identification of no eligible studies for inclusion.

Following the literature search, we imported the citations of our literature search into Rayyan software [21] to remove duplicates. Subsequently, two authors (P.C.N. and R.A.N.) conducted title and abstract screening using the research objectives, inclusion, and exclusion criteria as a guide. A third author (O.E.A.) resolved conflicts after a robust discussion with the other two authors. Next, we retrieved full texts for all studies and conducted a full‐text screening using the guidelines described earlier. Conflicts at this stage were resolved through a consensus with the other two authors (P.C.N. and R.A.N.). All potential issues and conflicts at the screening stages were adequately addressed. We did not contact authors for additional information or clarification on all the screened studies [22]. No study was found eligible for inclusion in this scoping review. The flow of the study selection procedures is summarized in Figure 1.

### 2.6 Quality Assessment of Included Studies

We intended to appraise the quality of any study included in this scoping review, using the Mixed Methods Appraisal Tool (MMAT) 2018 [23].

The MMAT is a standardized tool designed to critically evaluate empirical research across a range of study types, including qualitative studies, quantitative designs (randomized controlled trials, non‐randomized, and descriptive studies), and mixed methods research. The tool comprises a structured framework that begins with two universal screening questions applicable to all study types, followed by five criteria tailored to each specific design. Each criterion was scored as “Yes” (1 point), “Can't tell” (0.5 points), or “No” (0 points), depending on the clarity and completeness of the information provided.

We intended to award a cumulative score ranging from 0 to 7 for each of the included studies. Based on this scoring system, we intended to classify our scoring as “below average” (score < 3.5), “average” (score 3.5), or “above average” (score > 3.5). However, this intended quality appraisal could not be done because no study was found eligible for inclusion into this scoping review.

### 2.7 Data Charting

Data charting was intended to be done in this scoping review. Due to this intention, members of the research team were asked to submit preliminary data extraction templates based on the objectives and goals of this review. Subsequently, the lead author reviewed all submissions and developed an integrated data extraction sheet, which was presented to the research team. The research team critically reviewed the final submission and jointly approved this version after reaching a consensus on all elements of the data extraction theme.

The following information were intended to be extracted from all included studies: study title, lead study author, study location/country in Africa, study design, study sample size, study aims or objectives, study focus (mobile health applications, mobile phone‐based short messaging services, wearable portable devices etc.), type or theme of MHM, sample characteristics (age, sex, race or ethnicity), participant recruitment method (household survey, hospital survey, clinic survey etc.), and study outcomes (primary and secondary). However, this intended data charting procedure could not be done because no study was found eligible for inclusion into this scoping review.

## 3 Collation, Summarization, and Reporting of Results

A narrative synthesis approach was the intended approach that was to be used to collate, summarize, and report the data that would be charted from any study included in this scoping review. The intention was to organize all charted data into themes following an iterative procedure to group the information into broader categories, leveraging the objectives of this review. However, this intended data collation and summarization, and the thematic presentation of summarized data, could not be done because no study was found eligible for inclusion into this scoping review. Hence, only the results obtained from the literature search and selection processes were presented.

## 4 Results

The database search generated 687 records (PubMed = 119; SCOPUS = 561; AMED—The Allied and Complementary Medicine Database = 0; CINAHL Ultimate = 7; Dentistry and Oral Sciences Source = 0; SPORTDiscus with Full Text = 0; APA PsycArticles = 0; Psychology and Behavioral Sciences Collection = 0; and APA PsycInfo = 0). After removing 118 duplicates, a total of 569 non‐duplicate records were available for screening. Titles and abstracts of these records were independently assessed by three reviewers, aided by the predefined eligibility criteria. After title and abstract screening on Rayyan software, the reviewers could not identify any eligible studies on mHealth interventions for menstrual hygiene in Africa; this was primarily due to the non‐relevance of the population, context, or outcomes of interest. Consequently, no article was finally included in this scoping review (Figure 1).

## 5 Discussion

The focus of this scoping review was to identify and map relevant original literature that investigated the role of mHealth interventions in improving menstrual hygiene in Africa through a comprehensive search of peer‐reviewed publications. Although 687 records were retrieved from the database search, none of them satisfied the eligibility criteria after duplicate removal and thorough title and abstract screening. This screening outcome provides clear evidence that a significant gap exists on this subject. This is a notable finding on the current state of scientific research on menstrual hygiene in Africa, despite its public health importance.

The absence of eligible studies in this review is of concern, considering the number of challenges associated with menstrual hygiene in Africa, such as inadequate menstrual health knowledge, limited access to sanitary products, inaccessibility of water, sanitation, and hygiene (WASH) facilities, and widespread stigma [2, 24, 25, 26, 27]. Moreover, menstrual hygiene is influenced by poverty, gender inequality, cultural taboos about menstruation, and social discrimination [2, 27].

An opportunity to address these issues is through mHealth interventions, which have been effective in tackling diverse public health issues across low‐ and middle‐income (LMIC) settings. For instance, mHealth interventions have been utilized to resolve challenges associated with maternal and child health, HIV/AIDS, chronic non‐communicable diseases, and sexual and reproductive health [28, 29, 30]. Hereby, there is a track record for the feasibility, usability, and acceptability of mHealth technologies [29]. However, our findings indicate that mHealth pathways have not been exploited around menstrual hygiene in Africa.

One possible reason for the limited evidence is that menstrual hygiene has only gained recognition as a significant public health and human rights issue in recent years [31]. Even at that, it is not yet universally acknowledged as a health priority [32]. Its current recognition within development discourse is marginal, as it is not fully integrated into global development priorities [31]. Another contributing factor is the novelty and contextual specificity of mHealth interventions for improving menstrual hygiene in Africa, which is the focus of the current review. The lack of published research exploring the use of mHealth technologies to support menstrual hygiene practices may be due to the novelty of such interventions in the African region. Similar challenges have been noted in other reviews, mostly in low‐ and middle‐income countries where published research work and formal documentation of emerging, innovative, or under‐researched subjects may be limited [33].

It is also possible that relevant research, reports, or ongoing projects remain unpublished or not yet in peer‐reviewed outlets. Additionally, there is limited access to information and communication technologies in many African countries, including gaps in mobile phone ownership and digital literacy [34]. This may have presented challenges to the implementation and research on mHealth interventions on menstrual hygiene on the continent. Although menstrual hygiene is a vital element of women's and adolescent girls' health [2], currently, menstrual health is not integrated into the larger health and education policies in several countries [35]. In spite of the fact that the menstrual cycle involves emotional, physical, and social changes, in many African cultures, taboos, stigma, and lack of appropriate education leave many young women without proper menstrual hygiene knowledge, forcing them to rely on traditional practices [2]. This leads to school absenteeism, limits education and its outcomes, hinders social and economic participation, and poses long‐term reproductive risks and poor health outcomes [2, 27, 35, 36].

Therefore, the research gap on mHealth for menstrual hygiene represents missed opportunities to exploit mobile technological tools to provide the much‐needed education and support for the affected population. The findings of this review further reveal the potential misalliance between people's experiences and needs, evidence needs of policymakers and practitioners, and the current scope of published literature on menstrual hygiene in Africa. Hence, there is a need for collaboration between the community, researchers, public health practitioners, and policymakers. This is to ensure that future studies align with real‐world priorities and needs.

## 6 Conclusion

Despite the abundance of evidence reporting widespread adoption of mobile technology and challenges with menstrual hygiene, such as inadequate WASH facilities, limited product access, and stigma affecting millions across Africa, this scoping review identified no eligible empirical evidence examining the role of mHealth in addressing menstrual hygiene on the continent. This review stresses a critical research gap, considering that mHealth has proven effective in other areas such as maternal health and HIV. This may be attributed to recent recognition of menstrual health as a public health priority, cultural taboos limiting discourse, and possible unpublished or non‐peer‐reviewed work on the subject.

The outcome of this review has implications for research, policy, and practice. First, there is a need to prioritize studies that will focus on designing, implementing, and evaluating mHealth technologies targeting menstrual hygiene in Africa to fill the knowledge gap. Second, stakeholders need to integrate menstrual hygiene into sexual and reproductive health policies/frameworks, and also utilize mHealth for solutions in low‐resource settings. Lastly, the importance of multi‐sectoral collaborations across levels of government, non‐governmental organizations, tech experts, and others cannot be stressed enough. Such collaborations will help to initiate mHealth interventions, address barriers like digital literacy, cultural issues, and stigma for improved menstrual hygiene and health outcomes.

## Author Contributions

**Kafayat Aminu:** conceptualization, data curation, formal analysis, investigation, methodology, project administration, resources, software, supervision, validation, visualization, writing – original draft, writing – review and editing. **Precious Chika Nnannah:** conceptualization, data curation, investigation, methodology, project administration, resources, software, supervision, validation, visualization, writing – original draft, writing – review and editing. **Oluwatobi Emmanuel Adegbile:** conceptualization, data curation, investigation, resources, software, validation, writing – original draft. **Adetayo Olorunlan**a**:** investigation, resources, writing – original draft. **Yovanthi Anurangi Jayasinghe:** data curation, formal analysis, investigation, resources, validation, writing – original draft. **Ugochukwu Anthony Eze:** writing – original draft. **Afeez Abolarinwa Salami:** investigation, methodology, resources. **Emeka Benjamin Okeke:** investigation, resources, writing – original draft. **Olubukola Omobowale:** resources, writing – original draft. **Michael Renfrew:** resources, writing – original draft. **Rita Amarachi Nwebo:** conceptualization, data curation, investigation, methodology, project administration, resources, software, supervision, validation, visualization, writing – original draft, writing – review and editing. **Kehinde Kazeem Kanmodi:** conceptualization, data curation, formal analysis, funding acquisition, investigation, methodology, project administration, resources, software, supervision, validation, visualization, writing – original draft, writing – review and editing.

## Funding

The authors received no specific funding for this work.

## Ethics Statement

The authors have nothing to report. This study did not collect data from human or animal subjects but from an open research repository.

## Conflicts of Interest

Kehinde Kazeem Kanmodi is an Editorial Board member of *Health Science Reports* and co‐authors of this article. To minimize bias, he was excluded from all editorial decision‐making related to the acceptance of this article for publication. The other authors declare no conflicts of interest.

## Transparency Statement

The lead authors, Kafayat Aminu, Rita Amarachi Nwebo, and Kehinde Kazeem Kanmodi, affirm that this manuscript is an honest, accurate, and transparent account of the study being reported; that no important aspects of the study have been omitted; and that any discrepancies from the study as planned (and, if relevant, registered) have been explained.

## Supporting information


**Table S1:** Search string for PubMed database search. **Table S2:** Search string for SCOPUS database search. **Table S3:** Search string for other database (AMED – The Allied and Complementary Medicine Database, CINAHL Ultimate, Dentistry and Oral Sciences Source, SPORTDiscus with Full Text, APA PsycArticles, Psychology and Behavioral Sciences Collection, and APA PsycInfo) search via EBSCO interface.

## Data Availability

The authors confirm that the data supporting the findings of this study are available within the article and its Supporting Information.
